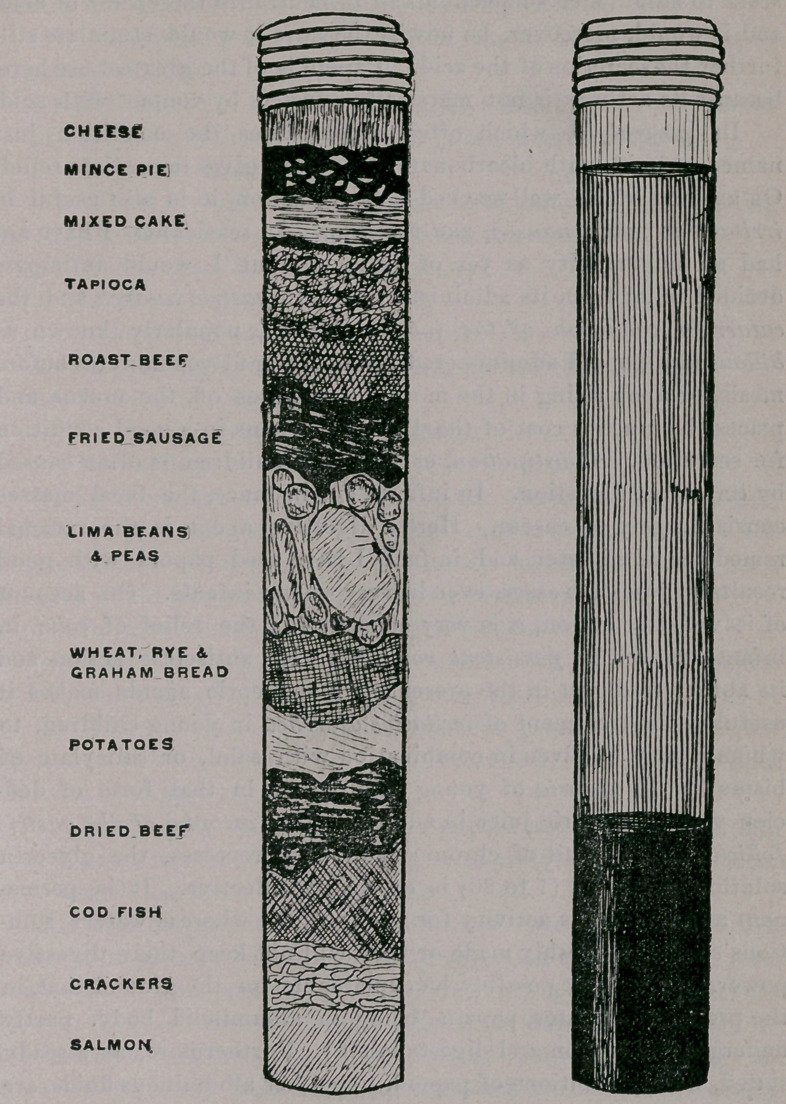# On the Digestive Ferment of the Carica Papaya in Gastro-Intestinal Disorders

**Published:** 1892-09

**Authors:** Frank Woodbury

**Affiliations:** Professor of Clinical Medicine in the Medico-Chirurgical College at Philadelphia, etc.


### Selection.

### ON THE DIGESTIVE FERMENT OF THE CARICA PAPAYA IN GASTRO-INTESTINAL DISORDERS.

By FRANK WOODBURY, A. M., M. D.,
Professor of Clinical Medicine in the Medico-Chirurgical College at Philadelphia, etc.

During the past year, having devoted considerable attention to the clinical applications of papoid (papain Finckler), especially in digestive disorders, I have had the satisfaction of witnessing a number of very interesting results, to which I wish briefly to direct attention. The successful application of physiological data must be my excuse for again directing attention to a remedy which has been studied by such eminent investigators as Wurtz and Bouchut, Finckler, Rossbach, Roy, and Wittmack, and one, furthermore, the physiological and therapeutical actions of which, at the present day, may be regarded as pretty fully established. If I have little of novelty to offer as regards the agent employed, I may at least point out very briefly some of its clinical uses and the conditions of its successful employment. If I accomplish this modest task, the labor will not be in vain, since success in therapeutics depends upon pharmaceutical preparation and mode of administration, in many instances, as much as it does upon the selection of the proper remedy.

There were two considerations that especially led me to study the clinical applications of the juice of the papaw to disorders of digestion. The first was the relatively large number, both in private practice and clinical service, of patients otherwise enjoying good health, but complaining of digestive disorders. The second was the following statement of Lauder Brunton's, which I encountered some years ago :

"In the West Indies, a tough beefsteak is rendered tender by rubbing it with the juice of a fresh papaw fruit, which contains a ferment, papain, having an action very much like the trypsin of the pancreas."[1]

The line of argument that would naturally be followed by the mind after receiving such a statement would be this: "A tender beefsteak is more easily masticated and digested than a tough one; consequently, an agent possessing the power of making this change must be of considerable value as an aid to digestion when weakened from any cause." Before considering the therapeutics of this unique remedy, however, I may briefly summarize its physiological actions and other properties.

For more than a century it has been known that the milky juice of the papaw has the power of softening meat, and it is still in general use for this purpose by the natives in the West Indies and some districts of South America. Indeed, it is stated that at Quito, where, on account of the elevation, water boils at too low a temperature to cook the meat tender, the juice of the papaw is a culinary necessity. The South America melon-tree, papaw, or *Carica papaya*, is indigenous to the tropical portion of this continent, but is easily cultivated in other warm countries and in hot-houses. It bears a large melon-shaped fruit, turning yellow when ripe, containing a great many seeds, the pulp having a not disagreeable taste, except that, according to Rossbach, it slightly suggests the odor of turpentine. The trunk of the tree, the leaves, and the fruit, all contain a large quantity of a milky juice, which rapidly undergoes a fermentive process and separates into two equal portions, a fluid and a semi-solid. The latter, which, when dried, is soluble with difficulty, received the name of "papayotin" from Peckolt, of Rio Janeiro. By the addition of sugar or glycerin and a few drops of peppermint oil, the fermentation may be prevented and the juice retain its active properties for some time. If alcohol be added to to this milky juice, the digestive ferment will be precipitated, and to this the name of "papain" was given by Wurtz and Bouchut, who, in 1879, made the first thorough investigation of this agent. Analyses made by Wurtz, however, showed remarkable discrepancies in chemical composition and physical properties. Rossbach subsequently suggested that this variation might be due to the presence of peptones, which he succeeded in separating by dialysation.

1. On Disorders of Digestion, their Consequences and Treatment. Lettsomian Lectures. By T. Lauder Brunton. London, 1886, p. 54.

He found the pure papain approximating the general constitution of albuminoid bodies in its composition, as, in addition to carbon, hydrogen, and nitrogen, it contains about 2.61 per cent. of sulphur. Its solutions, however, do not coagulate on boiling, but are liable in a few days, if left undisturbed, like other albuminous solutions, to become foul with vibrios and bacteria. From a clinical and therapeutic standpoint, the following chemical reactions possess some interest:

Hydrochloric acid, added to a solution of papain, causes a heavy precipitate, which, however, is soluble in an excess of acid. The same result is seen from the addition of nitric acid or metaphosphoric acid, but not from ordinary phosphoric acid or acetic acid. Corrosive sublimate does not cause any precipitate, or at the most a slight cloudiness, which afterward becomes more pronounced; on the contrary, if heat be applied, a heavy flocculent precipitate takes place.—(Rossbach.)

Professor Finckler, of Bonn, devised a process of purification of papain which yields a superior product, and free from albuminates and peptones to a degree not hitherto attained. This papain Finckler, or "papoid," as in this country it is known commercially, is a fine cream-white powder, almost devoid of odor and taste, freely soluble in both water and glycerin, and claimed to be of uniform digestive activity. If it should be introduced into our national pharmacopeia, analogy would require a change of termination, and probably "caricinum" or "caricin" would be the more acceptable designation for the pure product than either papoid or papain, which is liable to be confounded with other preparations from the juice, which are of much less digestive power and contain a variable proportion of peptones and other impurities.

The physiological actions of papoid as a digestive agent have been thoroughly established. It acts upon albuminoids, hydrating them and converting them ultimately into peptones, as fully demonstrated by George Herschell.[1] It converts starch with great promptness, the ultimate product being maltose. It emulsifies fats. Moreover, Herschell declares that it has a direct tonic action upon the stomach, stimulating the secretion of gastric juice or pepsinogen. Papoid, according to the same authority, is distinctly antiseptic in its action, and prevents abnormal fermentive processes from taking place in the stomach and intestines. An important point is, that it can be given in conjunction with true antiseptics,

1. Indigestion: A manual of the Diagnosis and Modern Treatment of the Different Varieties of Dyspepsia. By George Herschell, M. D., London. Page 140, London, 1892.

such as salol, when necessary, without its digestive action being checked; even corrosive sublimate in dilute solutions does not interfere with its digestive powers. It acts at all temperatures, but attains its maximum activity at a temperature of 130° F. In several important points it differs from pepsin. Papoid acts best in alkaline solution, but also can work in fluids with an acid or neutral reaction; pepsin requires an acid solution. Papoid is freely soluble and is most active when in concentrated form; pepsin requires free dilution. Herschell also points out the greater digestive power possessed by papain Finckler than either pepsin or pancreatin, and states that "it can be used when pepsin is contra-indicated or powerless." Finally, it should be stated that papoid has no action upon living tissues, and is positively innocuous when swallowed in any quantity that is likely to be administered.

Therapeutically, confining these remarks strictly to digestive disorders, papoid is useful when digestion has been overtaxed, or when the secretion of gastric juice is absent or deficient. Experiments of my own and others, made with the kind assistance of Mr. F. B. Kilmer, in Messrs. Johnson & Johnson's laboratory, at Newark, have satisfied my mind of the remarkable digestive activity of papoid. For instance, in one of the experiments referred to, portions of the constituents of a hearty dinner of bread, meat, potatoes, peas, mince-pie, and other substantials were placed in a large test-tube and treated with papoid and bicarbonate of sodium and a small amount of water. The result was very satisfactory indeed; the meat rapidly softened and the other ingredients gradually disintegrated, forming a pultaceous mass, which finally separated into a grumous sediment and an overlying albuminous, dark-colored liquid. [The artist has endeavored to celebrate this victory of papoid in the accompanying illustration, (see Fig., p. 101,) the only criticism upon which that can be offered is that it is, if anything, rather too graphic.]

Since papoid acts in alkaline solutions even better than in acid media, it is evident that it is specially useful where there is *indigestion due to deficient secretion of gastric juice* or of hydrochloric acid (achlorhydria). In such cases, the administration of an alkaline solution of papoid favors gastric digestion both directly and indirectly; first, by digesting albuminates and softening masses of food, and, secondly, by the action of the papoid in stimulating the secretion of the pepsin glands, while the alkali induces the secretion of a more acid gastric juice. Moreover, it retards the fermentation of the undigested masses of food in the stomach and prepares them for intestinal digestion. In fact, in such cases, a compressed pill of papoid, bicarbonate of sodium, and extract of nux vomica has given me excellent results. In the contrary case, *where there is an excess of hydrochloric acid*, and where the stomach contents poured into the duodenum are so acid that they prevent the action of the trypsin, papoid prevents duodenal indigestion by taking the place of the pancreatic ferment. As Herschell points out, it is obviously of no use to give pancreatin by the mouth, as it is at once destroyed by the acid of the stomach, and it is practically impossible to administer sufficient alkali to neutralize the excess of acid, and it would, moreover, be unwise, because it would stimulate still further the secretion of the acid. Papoid is of the greatest use here, because its activity is not materially affected by contact with acid.

In *gastralgia*, which often accompanies the condition just named, papoid, with bicarbonate of sodium, gives immediate relief. On account of its well-marked sedative action, it is also useful in *irritable stomach, nausea, and vomiting.* In seasickness I have not had an opportunity as yet of using it, but I would anticipate decided relief from its administration. In *gastric catarrh* and the *catarrhal condition of the intestinal tract*, popularly known as *biliousness*, papoid administered in hot water fifteen minutes before meals, or upon rising in the morning, cleanses off the mucus and places the mucous coat of the digestive organs in a good condition for secretion. *Constipation*, especially in children, is often caused by imperfect digestion. In infants, for instance, the fecal masses consist largely of casein. Here a digestive agent is the rational remedy to administer, and, in fact, I have used papoid with good results in just such cases, even in very young infants. On account of its sedative action, it is very efficient for the relief of *colic* in infants, as well as *persistent vomiting.* Its antiseptic action and its ability to digest in the presence of antiseptic agents makes it useful in the treatment of *irritative diarrhea* in young children, to whom it may be given in combination with salol, or salicylate of bismuth. In *apepsia* of young children, or in that form of deficiency of the gastric juice in adults, due to *atrophy of the gastric follicles* as the result of chronic catarrhal processes, the glycerin solution of papoid (1 to 20) is especially effective. It is permanent and retains its activity for a long time, whereas watery solutions should be freshly made or they will not keep their digestive power. (This may possibly be explainable on the ground that, in the presence of water, papain, being an albuminoid body, partly undergoes hydration and digests itself.) Furthermore, as already stated, watery solutions of papoid, like other albuminous fluids, are apt to become attacked by bacteria and undergo decomposition after standing for several days.

The uses of papoid, in treating disorders of the digestive organs, may be summarized somewhat as follows :[1]

---

1. This table agrees in the main with that of Herschell, *loc. cit.*

1. In actual or relative deficiency of the gastric juice, or its constituents.

(*a*) Diminished secretion of gastric juice as a whole; apepsia; anemia and deficient blood supply; wasting diseases.

(*b*) Diminished proportion of pepsin; atonic dyspepsia; atrophy of gastric tubules.

(*c*) Diminution of hydrochloric acid; achlorhydria; carcinoma.

(*d*) Relative deficiency of gastric juice; overfeeding.

2. In gastric catarrh.

(*a*) Where there is tenacious mucus to be removed, thus enabling the food to come in contact with the mucous membrane.

(*b*) Where there is impaired digestion.

3. In excessive secretion of acid.

To prevent duodenal dyspepsia.

4. In gastralgia, irritable stomach, nausea or vomiting.

5. In intestinal disorders.

(*a*) In constipation due to indigestion.

(*b*) In diarrhea, as a sedative.

(*c*) In intestinal worms. (This claim[1] the writer has not personally verified, but as the intestinal mucus which shields the worms is removed by papoid, it is easily understood that their removal would naturally result after its administration.)

6. In infectious disorders of the intestinal tract.

(*a*) Where there is abnormal fermentation; by its antiseptic action, which may be heightened by combination.

(*b*) Where there are foreign substances present, its detergent effect may be utilized in cleaning out the *débris* from the intestinal contents by digestion.

7. In infantile indigestion; here papoid not only readily peptonizes cow's milk, but the resulting curds are also soft and flocculent, resembling those of breast milk.

The dose of papoid, ordinarily, is one or two grains, but five grains or more may be used, the only objection being that of useless expense and waste, except where very prompt effects are desired, in which case even larger doses of the remedy may be administered. In case of obstruction of the esophagus by an impacted piece of meat and gristle—such as has been recently reported—a paste of papoid and water with some soda would produce softening in a very few minutes.—*New York Medical Journal*, July 30, 1892, p. 115, *et. seq.*

1. Descourtely and Tussac, *Flore des Antilles*, mentioned by Rossbach, *loc. cit.*

## Figures and Tables

**Figure f1:**